# The Difference in Scar-Related Quality of Life in Open Versus Closed Septorhinoplasty

**DOI:** 10.7759/cureus.40541

**Published:** 2023-06-16

**Authors:** Omar Braizat, Nasrin Jafarian, Dana Al-Majid, Mohammed El-Debs, Mahmoud Althalathini

**Affiliations:** 1 Department of Plastic and Reconstructive Surgery, Hamad Medical Corporation, Doha, QAT; 2 Department of Head and Neck Surgery, McGill University, Quebec, CAN

**Keywords:** patient reported outcome measure, health-related quality of life, patient satisfaction, scar, rhinoplasty

## Abstract

Introduction: The open and closed techniques are the main surgical techniques to perform septorhinoplasty. Although the open technique offers a better view of the pertinent anatomy and facilitates surgical access, it creates an external scar that could affect patients’ satisfaction and quality of life (QoL). This study aims to compare the open and closed techniques using the SCAR-Q patient-reported outcome measure.

Methods: In this retrospective study, we have included patients who had their nasal surgery one year ago, in the period between April 2020 and April 2021. The SCAR-Q assessment tool to study patients’ satisfaction with appearance, symptoms, and psychological impact of open and closed septorhinoplasty techniques.

Results: A total of 77 patients were included in this analysis. Of these, 39 (50.6%) patients underwent a closed septorhinoplasty, and 38 (49.4%) patients underwent an open approach. The mean (SD) age was 29.6 (8.1) years, and most patients were females (59.7%). The overall SCAR-Q questionnaire responses were very positive across all scales in our cohort, the median (IQR) scores were 91.0 (73.0-100.0) for the appearance scale, 89.0 (70.0-100.0) for the symptoms scale, and 100.0 (87.0-100.0) for the psychological impact scale. However, we have found no differences in SCAR-Q scores regarding appearance, symptoms, and psychological impact between open and closed septorhinoplasty.

Conclusion: We have found no significant differences in QoL between open and closed techniques of septorhinoplasty. Larger studies are needed to further validate this finding.

## Introduction

Septorhinoplasty has medical and cosmetic indications. Two main surgical techniques exist for approaching septorhinoplasty; the closed endonasal technique where the scar is hidden inside the nose and the open technique where an external columellar scar is visible [1]. Using the open technique at the core joining the two endonasal incisions in the columella, this small joining incision grants the surgeon a better appreciation of the cartilaginous infrastructure of the nose, which is sacrificed in the closed technique [2]. By revealing the underlying nasal anatomy, surgeons are better able to thoroughly analyze nasal asymmetry or structural problems, which results in enhanced precision and direct visualization of the effect of any surgical manipulation [3]. The main argument against the open technique is the external scar and its potentially detrimental effect on the quality of life (QoL) of patients [4]. However, many authors advocate for the open technique as they do not regard the columellar scar as such a significant cause of morbidity [2,5].

Satisfaction with the scar and its effect on the QoL of patients is an integral part of evaluating the final outcome of the septorhinoplasty procedure. Patient-reported outcome measures (PROMs) are common tools for evaluating such subjective variables [6,7]. Multiple PROMs exist, however, the SCAR-Q is a novel and precise PROM that has been validated for assessing how a scar impacts a patient's QoL [8,9]. It has not been confined to any particular region of the body and has been tested on a variety of aetiologies, including burns, surgery, and trauma [6,10]. Choo et al. discovered that SCAR-Q has the most rigorous validation process and strong reliability, and is a suitable tool for directing surgical management based on the QoL when compared to five other PROMs [6]. SCAR-Q has also been translated and validated into the Arabic language and will be applied to our patients in both languages [11].

Various studies have already attempted to evaluate the burden of columellar scars on QoL, notably in the Arabic race. In a study involving 600 Arab patients, Foda et al. concluded that open nasal surgery can be conducted on Arab patients safely and with minimal scarring [12]. However, a second prospective study conducted on 50 Saudi Arabian patients indicated that 22% of septorhinoplasty patients will have poor columellar scars [13]. None of these studies compared this burden between patients undergoing the closed and open technique and none of them used the newly developed and reliable SCAR-Q questionnaire. In this study, we aim to investigate the burden on QoL of patients who underwent open septorhinoplasty versus the closed endonasal approach using the SCAR-Q. Columellar scars, in general, tend to heal well and are usually barely noticeable, thus we predict that the open technique should not cause a significant deterioration in scar QoL.

## Materials and methods

Study design and participants

This study has a retrospective cohort design. After obtaining ethical approval from the Medical Research Committee (Approval number: MRC-01-21-919), electronic patient records were used to generate a list of patients who had their nasal surgery more than one year ago (01/04/2021-01/04/2022). A period of at least one year after surgery was implemented to allow for scar maturation. The search generated a list of 150 patients between the age of 18 and 65 who had open and closed (endonasal) septorhinoplasty. Due to COVID-19 social gathering restrictions, all 150 patients were approached via telephone by plastic surgery or ENT residents. A previously designed telephone script was followed and started by confirming the identity of the potential participant through their name and health card number. The surgeon then proceeded to explain the study procedures, duration, any potential risks, and the measures taken to maintain confidentiality.

Participation was voluntary, patients accepting to join the study signed an electronic consent form. Then a link containing the SCAR-Q questionnaire was sent directly to the patient either in English or Arabic depending on their preferred language. Important demographic data related to scarring patterns like age, sex, smoking status, ethnicity, and history of excessive scarring. The method of skin closure and characteristics of suture material used were also collected from the chart of consenting patients. None of the patients applied any anti-scarring treatment except for avoiding sun exposure for the first six months after the operation. Patients known to be keloid or hypertrophic scar formers or diagnosed with any psychiatric illness were excluded from the study.

Study materials

The SCAR-Q questionnaire was originally developed in the English language by authors at McMaster University [9]. It was later translated into several languages. We will be using the original English version and the translated and validated Arabic version [11].

Data collection

The questionnaires filled out by the participants along with the demographic data collected were all compiled into an Excel spreadsheet. Any patient identifiers were then removed from the data, and the score for each one of the three SCAR-Q subscales along with the overall score was calculated and added. The data was then downloaded into a USB drive and stored in a secure cabinet.

Statistical analysis

Descriptive measures included means with standard deviations (SD) for the continuous data. Categorical data were presented by frequencies and percentages (%). Tables and figures were used to visualize the data. For analytical purposes, the age variable was further divided into three groups based on the percentiles, 18-22 (<25%), 23-36 (25%-75%), and 37-50 (>75%). Continuous data were compared using the student’s t-test and one-way analysis of variance (ANOVA) in normally distributed variables, and the Mann-Whitney U and one-way Kruskal-Wallis test if not normally distributed according to the Shapiro-Wilk test of normality. The pairwise deletion method was used when dealing with missing data, considering it was missing completely at random. Statistical significance was considered at a two-sided p-value of ≤ 0.05. All data analyses were performed using Statistical Product and Service Solutions (SPSS) (IBM SPSS Statistics for Windows, Version 26.0, Armonk, NY) [14].

## Results

A total of 77 patients were identified at our institute, of which 50.6% (39/77) had undergone closed septorhinoplasty and 49.4% (38/77) had undergone open septorhinoplasty. Our cohort had a mean (SD) age of 29.6 (8.1) years and a female predominance of 59.7% (46/77). The majority were non-smokers (84.4%, 65/77), single (63.2%, 48/77), and of Qatari nationality (67.5%, 52/77). Lastly, six patients (7.8%) had additionally undergone an alar reduction surgery, and five (6.5%) required revision surgery. All columellar incisions were closed using interrupted 5.0 nylon sutures and 4.0 polyglactin sutures were used for the internal nasal incisions. Patients’ characteristics are summarized in Table 1.

**Table 1:** Socio-demographic characteristics of participants

Patient characteristics	Overall (N=77)
Age	
Mean (SD)	29.6 (8.1)
18-22 years	16 (20.8%)
23-36 years	43 (55.8%)
37-50 years	18 (23.4%)
Gender	
Male	30 (39.0%)
Female	47 (61.0%)
Smoking	12 (15.6%)
Marital status	
Single	49 (63.6%)
Married	28 (36.4%)
Nationality	
Qatari	52 (67.5%)
Non-Qatari	25 (32.5%)
Ethnicity	
Arabs	73 (94.8%)
Non-Arabs	4 (5.2%)
Type of surgery	
Open	38 (49.4%)
Closed	39 (50.6%)
Alar reduction surgery	6 (7.8%)
Revision surgery	5 (6.5%)
SCAR-Q score, Mean (SD)	255 (53.7)
Appearance	83.0 (21.0)
Symptoms	83.2 (18.0)
Psychosocial impact	89.1 (22.4)

Appearance, symptoms, and psychological impact of SCAR-Q

Scales

The overall SCAR-Q questionnaire responses were very positive across all scales in our cohort, the mean (SD) scores were 83.0 (21.0) for the appearance scale, 83.2 (18.0) for the symptoms scale, and 89.1 (22.4) for the psychological impact scale, and summing the three subscales scores resulted in a total mean (SD) score of 255 (53.7).

On an individual level, the appearance scale score did not differ between the closed and open septorhinoplasty, with a mean (SD) of 86.0 (18.8) vs 78.8 (23.0), respectively, p = 0.267. Patients who underwent alar reduction surgery scored higher scores than patients who did not, 85.8 (23.8) vs 82.3 (21.0), respectively, p = 0.591. On the contrary, patients who underwent additional revision surgery scored less than those who did not, 76.3 (28.3) vs 82.8 (21.0), respectively, but with no statistical significance as well (p = 0.938). For the demographics, the age and the gender variables did not impact the scores of the patients in our cohort, p = 0.658 and 0.836, respectively. Finally, Qatari patients had lower scores than their non-Qatari counterparts, 81.3 (20.8) vs 85.2 (22.0), p = 0.216 (Table 2).

**Table 2:** Scores of the appearance, symptoms, psychological impact dimensions, and the total SCAR-Q score

Variables	Appearance		Symptoms		Psychological		Total SCAR-Q	
	Score Mean (SD)	P-value	Score Mean (SD)	P-value	Score Mean (SD)	P-value	Score Mean (SD)	P-value
Age								
18-22 years	80.5 (19.6)	0.658	81.1 (21.0)	0.929	88.8 (18.7)	0.843	250.4 (45.2)	0.556
23-36 years	83.0 (20.2)		82.7 (17.2)		88.5 (23.2)		256.4 (53.5)	
37-50 years	83.2 (25.0)		84.4 (18.2)		89.4 (25.3)		256.9 (62.7)	
Gender								
Male	83.8 (18.8)	0.836	83.2 (17.7)	0.882	91.2 (18.3)	0.596	258.2 (43.6)	0.998
Female	81.6 (22.8)		82.5 (18.5)		87.1 (25.3)		253.3 (60.0)	
Smoking								
Smokers	86.7 (16.5)	0.605	82.9 (17.4)	0.837	96.9 (10.7)	0.138	266.5 (33.7)	0.667
Non-smokers	81.7 (21.9)		82.8 (18.3)		87.2 (23.9)		253.2 (56.6)	
Marital status								
Single	81.6 (19.6)	0.423	82.1 (17.8)	0.707	90.3 (18.5)	0.705	255.0 (45.3)	0.543
Married	84.1 (23.6)		83.9 (18.8)		86.3 (28.4)		254.3 (66.9)	
Nationality								
Qatari	81.3 (20.8)	0.216	81.6 (19.2)	0.441	87.4 (22.7)	0.183	251.2 (55.5)	0.261
Non-Qatari	85.2 (22.0)		85.3 (15.6)		91.8 (22.5)		263.8 (49.9)	
Type of Septorhinoplasty								
Open	78.8 (23.0)	0.267	82.8 (19.0)	0.655	86.6 (23.5)	0.319	250.9 (57.0)	0.530
Closed	86.0 (18.8)		82.7 (17.4)		90.9 (21.8)		259.5 (50.7)	
Alar Base Reduction surgery								
Yes	85.8 (23.8)	0.591	89.8 (22.8)	0.271	91.6 (18.9)	0.852	267.2 (64.8)	0.222
No	82.3 (21.0)		82.3 (17.8)		88.6 (22.9)		253.8 (53.4)	
Revision surgery								
Yes	76.3 (28.3)	0.938	83.0 (29.5)	0.491	86.0 (24.3)	1.000	259.0 (71.7)	0.486
No	82.8 (21.0)		82.8 (17.8)		88.9 (22.7)		254.4 (53.3)	

The symptoms scale scores also did not differ between the closed and open septorhinoplasty, with a mean (SD) of 82.7 (17.4) vs 82.8 (19.0), respectively, p = 0.655. Patients with an additional alar base reduction surgery or an additional revision surgery had higher scores than their counterparts, with a score of 89.8 (22.8) vs 82.3 (17.8) and 83.0 (29.5) vs 82.8 (17.8), respectively, but with no statistical significance for both, p = 0.271 and 0.491, respectively. The age, gender, and nationality variables did not impact the scale scores, p = 0.929, 0.882, and 0.441, respectively (Table 2).

**Figure 1:**
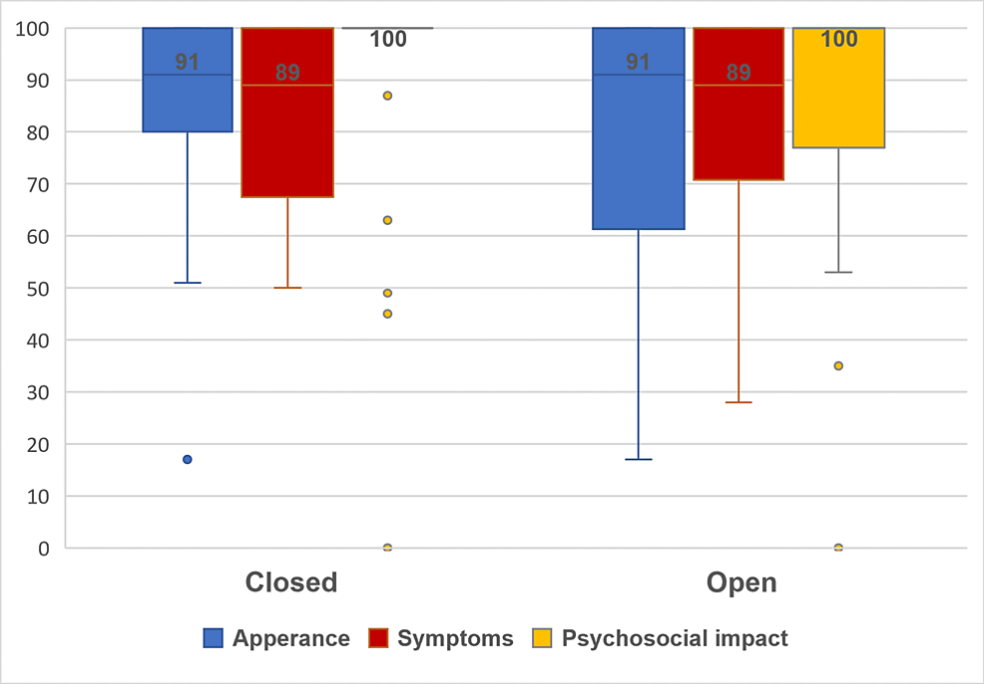
Closed vs open septorhinoplasty SCAR-Q scores

The psychological impact scale scores did not differ between the closed and open septorhinoplasty as well, with a mean (SD) of 90.9 (21.8) vs 86.6 (23.5), respectively, p = 0.319. The presence of an additional alar base reduction surgery or an additional revision surgery did not impact the mean scores, p = 0.852 and 1.000, respectively. Like the appearance and symptoms scales, the age and gender variables did not impact the scale scores, p = 0.843 and 0.596, respectively. Again, patients of Qatari nationality were more likely to have lower scores than non-Qatari patients, 87.4 (22.7) vs 91.8 (22.5), respectively, p = 0.183. (Table 2). Figure 1 visualizes the impact of the septorhinoplasty type (closed vs open) on the three scales.

Lastly, the summed total SCAR-Q score did not differ between the closed and open septorhinoplasty, with a mean (SD) of 259.5 (50.7) vs 250.9 (57.0), respectively, p = 0.530. Patients with an additional alar base reduction surgery or an additional revision surgery had higher total scores than their counterparts, with a total score of 267.2 (64.8) vs 253.8 (53.4) and 259.0 (71.7) vs 254.4 (53.3), respectively, with no statistical significance for both, p = 0.222 and 0.486, respectively. Differences in age, gender, and smoking status did not impact the total score in our cohort, p = 0.556, 0.998, and 0.667, respectively. Patients with Qatari nationality scored lower than non-Qatari counterparts, 251.2 (55.5) vs 263.8 (49.9), p = 0.261.

## Discussion

Health-related QoL has gradually become a cornerstone for assessing surgical outcomes in general and aesthetic surgical outcomes in specific [15]. According to the World Health Organization Quality of Life Group, a person's perception of their current state in life in relation to their goals, expectations, standards, and concerns is referred to as their health-related QoL. QoL is a broad and complex concept influenced by the person's physical health, psychological state, level of independence, interpersonal relationships, and their relationships to conspicuous aspects of their surroundings [16]. In practice, the range of QoL measurements used in applied research is less expansive, and it would probably be more accurate to refer to the majority of these measurements as PROM [17]. Since surgical care aims to improve patients' health, the effects of surgery on health-related QoL are arguably the most significant outcomes to measure in assessing the efficacy of surgical care. Several studies have shown that QoL after nasal surgery is significantly improved [18-20], and a recent systemic review performed by Wähmann et al. showed that functional-aesthetic rhinoplasty leads to a significant improvement in patients’ health-related QoL [21].

SCAR-Q [22] is a promising tool that was developed for both children and adults and is the first tool designed to be applied to all scar types including surgical, traumatic, and burn scars. It exhibits the most stringent content validation process and has a high degree of reliability. In contrast to other PROMs, which only address the clinical and psychosocial elements of scars, it covers scar appearance, symptoms, and psychosocial impact [6].

Our study attempted to determine the burden of the columellar scar on the QoL of patients undergoing septorhinoplasty. Thus, patients who underwent the closed endonasal approach represent the ideal control group to use against patients undergoing the open approach. The procedures in both techniques are very similar with the main difference being that the scar in the endonasal approach is hidden within the nose.

Our sample has a mean age is 29.6 years old with female predominance (59.7%), single marital status (63.2%), Qatari nationality (67.5%), ethnicity being middle eastern pre-dominantly (94.8%), brown (45.9%). Overall, the responses were positive across all scales with no difference between open and closed septorhinoplasty. However, no statistically significant differences in SCAR-Q scores regarding appearance, symptoms, and psychological impact between open and closed septorhinoplasty.

The literature that compares open and close septorhinoplasty in terms of QoL is scarce. One study was done by Kütük et al. to compare open and closed rhinoplasty with 45 patients in each group. They have used three assessment tools including the Nasal Obstruction Symptom Evaluation (NOSE), the Rhinoplasty Outcome Evaluation (ROE) for patient satisfaction, and the Derriford Appearance Scale (DAS-24) for psychological distress related to disfigurements, deformities, and aesthetic problems of physical appearance. In all assessments, they found no significant differences in scores between open and closed rhinoplasty [23], which was concordant with our results regarding QoL.

A systematic review has noted that reduced nasal sensation was reported by both patients who underwent open or closed rhinoplasty. However, patients with a reduced columellar sensation were only found in the open rhinoplasty group. But there was no consensus about which technique is better in terms of cosmetics and patient satisfaction [24].

Several PROMs exist and they all attempt to measure functional or aesthetic symptoms of patients undergoing rhinoplasty. According to a recent systematic review of 33 studies including 12 different measurement tools, the NOSE scale, FACE-Q, and SCHNOS were the most effective indicators of the outcome of cosmetic surgery [25]. The NOSE scale [26] focuses on nasal obstruction-related symptoms, and the FACE-Q Rhinoplasty module [22] consists of over 40 subscales measuring a variety of patient-reported outcomes of facial aesthetic treatment. And finally, the Standardized Cosmesis and Health Nasal Outcomes Survey aesthetic subscale (SCHNOS) [27] has both functional and aesthetic domains.

Scars and the symptoms they cause may impair a patient's QoL and physical, social, and psychological well-being [28,29]. The breadth of scar impact on a patient was commonly assessed through clinician-reported outcome (ClinRO) assessment measures. The Vancouver Scar Scale and Manchester Scar Scale [30] are two examples of traditional scar outcome assessments that emphasize the clinician's interpretation of the scar's physical characteristics. This clinician-reported tool measures observable signs [30], such as scar color or size, and potential physical indications of disease through clinical judgment and competence. Such measurements, however, do not account for symptoms or unobservable concepts, such as pain or QoL impact, which are only recognized by the patient [30]. To portray patients' perspectives, PROMs for scars are required [30].

This is the first study to our knowledge that assess the scar burden of scars on QoL between patients undergoing the closed and open septorhinoplasty techniques using the SCAR-Q questionnaire. The limitations include the retrospective nature of this study and the small sample size which limits the generalization of our findings and increases the risk of selection bias. Moreover, our study was not able to compare SCAR-Q scores among different Fitzpatrick skin types. This was not possible due COVID-19 pandemic and social distancing.

## Conclusions

In conclusion, the responses were positive across all scales with no statistically significant difference in SCAR-Q scores between open and closed septorhinoplasty groups. We believe our studies do provide reliable evidence to support the conclusion that open septorhinoplasty does not carry a significant burden over the scar-related QoL of patients when compared to closed septorhinoplasty. However, we also recommend conducting a multinational, multicentre prospective study that includes a larger number of patients using the SCAR-Q tool to further validate our findings.
